# Validation of SHOEBOX QuickTest Hearing Loss Screening Tool in Individuals With Cognitive Impairment

**DOI:** 10.3389/fdgth.2021.724997

**Published:** 2021-09-13

**Authors:** Andrew Frank, Serena Goldlist, Amy E. Mark Fraser, Matthew Bromwich

**Affiliations:** ^1^Bruyère Research Institute, Ottawa, ON, Canada; ^2^SHOEBOX Ltd, Ottawa, ON, Canada; ^3^Department of Otolaryngology- Head and Neck Surgery, CHEO, Ottawa, ON, Canada; ^4^Department of Otolaryngology- Head and Neck Surgery, University of Ottawa, Ottawa, ON, Canada

**Keywords:** hearing screening, memory loss, hearing impairment, mild cognitive impairment, aging, mild dementia, audiometry

## Abstract

**Objectives:** The aim of this study was to validate a novel iPad-based rapid hearing loss screening tool (SHOEBOX QuickTest) in individuals with cognitive impairment.

**Design:** Cross-sectional validation study.

**Setting:** Bruyère Research Institute, Ottawa, Canada.

**Subjects and Methods:** Twenty-five individuals with mild cognitive impairment (MCI) and mild dementia from the Bruyère Memory Program were included in this study. The study consisted of two components: (1) SHOEBOX QuickTest hearing screener and (2) a conventional hearing test (pure tone audiometry).

**Measurements:** Hearing was assessed at 1,000, 2,000, and 4,000 Hz separately for each ear. The agreement between hearing ability groupings (good vs. reduced) from conventional hearing test and SHOEBOX QuickTest was determined. Specifically, accuracy, sensitivity, specificity, as well as alignment between conventional thresholds and hearing threshold ranges.

**Results:** An overall accuracy of 84% was observed for SHOEBOX QuickTest, and a sensitivity and specificity of 100 and 66.7%, respectively. 72% ([95% CI], 60.0–84.1%) of conventional audiometry thresholds were within the pre-established 10 dB SHOEBOX QuickTest.

**Conclusion:** SHOEBOX QuickTest is a valid hearing loss screening tool for individuals with cognitive impairment. Implementing this iPad-based screening tool in memory clinics could not only aid in the timely diagnosis of hearing loss, but also assist physicians in providing a better assessment of cognitive impairment by ruling out hearing loss as a confounding variable.

## Introduction

Hearing loss has been identified as the most prevalent sensory disability in the world impacting approximately 432 million adults worldwide (1, 2). It has been associated with many adverse consequences such as social isolation (3), depression (4, 5), safety issues, decline in independence and reduced quality of life (6, 7). Despite its widespread presence, hearing loss is largely under-recognized and under-treated. It has been estimated that 67–86% of people who experience this disability do not use any form of hearing aid or other assistive technology (8). The World Health Organization (WHO) estimates an annual global cost of US$750 billion due to unaddressed hearing loss (1). This figure is expected to rise with the number of people facing this problem increasing globally. The economic impact is especially dire in countries with aging populations as prevalence of hearing loss increases with age (9). In the United States, it is estimated that two-thirds of people over 70 years old are affected (10). Typically, screening for hearing loss is not included in the battery of tests recommended by physicians for older adults.

In addition to hearing loss, dementia is a one of the major causes of disability among older adults worldwide (11). Dementia is an umbrella term for brain disorders leading to deterioration in cognitive function (11). At any given time, it is estimated that 5–8% of people aged 60 and older are suffering from dementia and 10 million new cases are added each year across the globe (11). The most common cognitive assessments are the Montreal Cognitive Assessment (MoCA), Mini-Mental State Exam (MMSE) and Mini-Cog (12). Currently, screening for hearing loss is not incorporated into these cognitive assessments even though it is estimated that over 60% of adults with cognitive impairment also have a hearing impairment (13). It has been proposed that hearing loss might be a marker for cognitive decline and could be a modifiable risk factor for dementia (14). Thus, ruling out hearing loss could assist physicians in providing a better assessment of cognitive impairment.

The gold standard for assessing hearing loss is pure tone audiometry (PTA) administered by trained audiologists (15). PTA assesses hearing sensitivity by determining hearing thresholds that are required to perceive a tone at least 50% of the time. Hearing thresholds are assessed at different frequencies ranging from 500 to 8,000 Hz and are then plotted on an audiogram to determine if patient's hearing levels are within normal limits (16). The limitation of PTA is that it requires access to specialized medical equipment and staff. However, despite the growing need for audiology professionals in our aging society, work force analyses have indicated that the demand for hearing specialists will outpace available capacity over the next few decades (17, 18). Therefore, there is a growing need to validate a reliable and effective tool to quickly screen older adults (including those who are cognitively impaired) for hearing difficulties to effectively triage them to specialists.

Alternative hearing loss screening methods that do not require specialized health care professionals or expensive medical equipment have been developed recently. These methods are more accessible as they are administered on personal computers (19), tablets (20) or smartphones (21, 22). Although these options provide a potentially more convenient and quicker assessment, issues concerning lack of validation and the effects of environmental noise (i.e., noise limiting) are unaddressed. SHOEBOX Audiometry (SHOEBOX Ltd, Ottawa, Canada) developed an approach to manage background noise levels by utilizing sound-attenuating headphones with their SHOEBOX QuickTest application. However, SHOEBOX QuickTest has yet to be validated in individuals with cognitive impairment.

To date, only one study has assessed hearing loss in cognitively impaired individuals using a screening method not administered by audiology professionals. Pletnikova and colleagues assessed the feasibility of using a tablet-based audiometer in individuals with cognitive impairment (23). Although it could reliably test 59% of the patients, lower cognitive assessment scores (i.e., MMSE) were associated with less reliable results. Furthermore, the study did not compare against a gold standard (i.e., PTA).

Even with the established association between hearing loss and cognitive decline combined with high rates of undiagnosed hearing loss in older adults, no studies have explored the suitability of a rapid hearing loss screening tool to screen for hearing loss in a population of individuals with cognitive impairment. Therefore, the aim of this study was to validate the usefulness of the iPad-based SHOEBOX QuickTest (SHOEBOX Ltd.) hearing screening application in a group of older individuals with cognitive impairment.

## Materials and Methods

### Participants

Twenty-five (25) individuals followed at the Bruyère Memory Program (Bruyère Continuing Care) were recruited into this study. All participants were diagnosed with mild cognitive impairment (MCI) or mild dementia. All patients meeting inclusion criteria were approached by a research staff member who explained the study and if interested, obtained informed consent. The experimental protocol was approved by Bruyère Ethics Review Board and participants were free to withdraw at any point. Demographic information regarding age, gender, and diagnosis, as well as the number of attempts to complete the SHOEBOX QuickTest are displayed in Table 1.

**Table 1:** Characteristics of participants with cognitive impairment.

**Characteristic**	***N* = 25**
**Sex**
Male	14 (56%)
Female	11 (44%)
**Age**	
50–60	1 (4%)
60–70	4 (16%)
70–80	16 (64%)
>80	4 (16%)
**Diagnosis**	
Mild dementia	6 (25%)
MCI	19 (76%)
**Number of attempts to complete** SHOEBOX QuickTest
1	18 (72%)
2	4 (16%)
3	3 (12%)

*Values are reported as number of participants (%)*.

### Hearing Assessments

**Figure 1:**
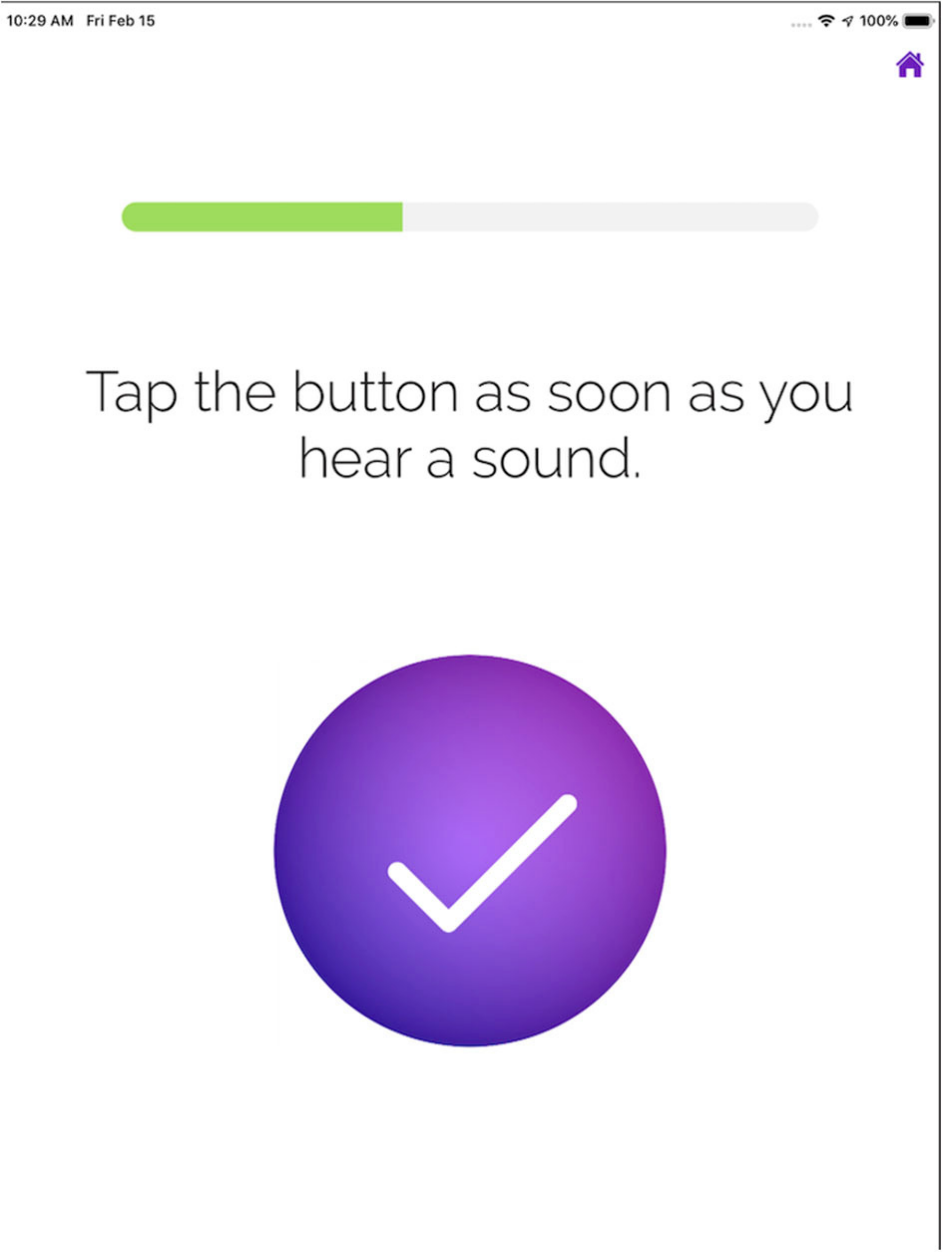
Display on iPad screen for participants to tap.

The iPad-based SHOEBOX QuickTest application was performed with calibrated sound-attenuating headphones. Testing took place in a quiet office at Élisabeth Bruyère Hospital. The complete test included two main components, a set of four questions followed by a series of pure tone presentations. The tone presentation component was performed separately for each ear (right then left ear) and included frequencies of 1,000, 2,000, and 4,000 Hz. Participants tapped the circle in the middle of the iPad screen (see Figure 1) to indicate that they had heard a sound. The presentation level (volume) varied algorithmically depending on the response or lack of response to the previous tone presentation. A starting volume of 70 dB HL (Hearing Level) was used. Tests were completed using the RadioEar DD450 transducers and results were not displayed to the patients. Pure tones (dB HL) were measured at each of these frequencies in each ear and categorized into 10 dB ranges (i.e., 0–10, 10–20, 20–30, 30–40, 40–50, 50–60, 60–70, 70–80). Participants were defined as having hearing loss if ranges obtained at any frequency (i.e., 1,000, 2,000 and 4,000 Hz) exceeded 30 dB HL (i.e., 30–40 dB HL) in any of their ears.

Following completion of the SHOEBOX QuickTest, participants underwent a conventional pure tone audiometry hearing assessment by an audiologist at the Ottawa Audiology Services Clinic, Ottawa, Canada (located at Élisabeth Bruyère Hospital) using circumaural headphones in a sound booth. The audiologist was blinded to the SHOEBOX QuickTest results. The SHOEBOX QuickTest was always conducted first, in order to disadvantage it against any potential sequential learning effect. Assessments included PTA as well as otoscopy performed on both ears. Results from testing at frequencies of 1,000, 2,000, and 4,000 Hz were used to obtain an overall assessment of hearing ability. Similar to SHOEBOX QuickTest, air conduction thresholds (dB HL) were measured at each of these frequencies to determine hearing loss. Participants were defined as having hearing loss from conventional pure tone audiometry if thresholds obtained at any frequency (i.e., 1,000, 2,000, and 4,000 Hz) exceeded 30 dB HL in any of their ears.

### Statistical Analysis

Presence of hearing loss was determined by the SHOEBOX QuickTest and conventional audiometry for each participant separately. Reduced hearing ability was defined as >30 dB and was entered in a 2 x 2 contingency table to determine the accuracy of SHOEBOX QuickTest in screening for hearing loss (sensitivity and specificity). Analysis was performed by a at Bruyère Research Institute using Statistical Program for the Social Sciences (SPSS) version 24 (SPSS Inc., Chicago, IL).

The pure tone thresholds obtained from conventional audiometry were compared to the corresponding ranges estimated in SHOEBOX QuickTest at each frequency and each ear. To determine the correlation between the two assessments, the proportion of pure tone thresholds obtained from conventional audiometry that fell within the estimated pure tone ranges from SHOEBOX QuickTest were computed with 95% confidence intervals. We also computed thresholds obtained by conventional audiometry that were within +/– 5 dB the range obtained by SHOEBOX QuickTest.

## Results

A total of 25 patients (mean age = 73.84 years) with MCI or mild dementia were tested using SHOEBOX QuickTest and conventional pure tone audiometry. All patients were able to complete the SHOEBOX QuickTest in three attempts without any assistance. Reasons for greater number of attempts required include not responding quickly enough, and not understanding the task on the first attempt (see Figure 1). Comparison of the SHOEBOX QuickTest to conventional pure tone audiometry is shown in Table 2. The sensitivity and specificity for SHOEBOX Quick Test were 100% (13/13) and 66.7% (8/12) respectively. Of the four patients who had conflicting results between conventional audiometry and the SHOEBOX QuickTest, all were identified as having hearing loss on the SHOEBOX QuickTest but good hearing on conventional audiology exam. The positive predictive value was 76% (CI 59–88%) and the negative predictive value was 100%, with an accuracy of 84%.

**Table 2:** Accuracy of SHOEBOX QuickTest in screening for hearing loss (>30 dB HL) in people with cognitive impairment.

	**Conventional Audiometry**	
SHOEBOX	≤ 30 dB HL	>30 dB HL
≤ 30 dB HL	8	0
>30 dB HL	4	13
Total	12	13

The measured pure tone threshold ranges computed using the SHOEBOX QuickTest were compared to the threshold obtained using conventional audiometry to determine correlation between the two assessments. 72% (95% [CI], 60.0%-84.1%) of conventional audiometry thresholds were within the 10 dB range of the SHOEBOX QuickTest. If we expanded the range by +/−5 dB given by SHOEBOX QuickTest, 89.33% (95% [CI], 98.34–80.33) of thresholds obtained by conventional audiometry were included.

## Discussion

The primary aim of this study was to validate the usefulness of an iPad-based hearing screener as a screening tool in patients with mild cognitive impairment (MCI) or mild dementia. Overall, the test was accurate with 84% of the tested population having matching results between conventional audiometry and the SHOEBOX QuickTest. SHOEBOX QuickTest was a highly sensitive assessment, correctly identifying every patient who had hearing loss (*n* = 13) as measured by an audiologist (sensitivity 100%). It is important to note that SHOEBOX QuickTest may be overly sensitive, identifying hearing loss in some individuals who do not have hearing loss (*n* = 4). Furthermore, despite the fact that SHOEBOX QuickTest was not intended to give exact thresholds, our results demonstrate that it is still relatively accurate at estimating hearing ranges. Lastly, three of the four patients with unknown cerumen accumulation were correctly identified using SHOEBOX QuickTest. Taking together, although SHOEBOX QuickTest identified some false positives, it can reliably screen for hearing loss in older adults with cognitive impairment.

In our study, all of participants were able to complete the self-administered screening tool within three attempts. This may be because we restricted our participants to those diagnosed with mild-stage cognitive impairment (i.e., mild cognitive impairment and mild dementia) and were likely less impaired than a previous study which included participants with more advanced dementia (23). Future studies should seek to validate the SHOEBOX QuickTest in participants with more advanced cognitive impairment.

There is a plethora of literature detailing the relationship between hearing loss and dementia. A recent article by Griffiths et al. proposed that hearing loss leads to an impoverished sensory environment that decreases stimulation and cognitive processing (24). The impoverished auditory input negatively alters brain structure and function which is a risk factor for the development of dementia. Using hearing aids has been associated with a reduced risk of developing dementia (25). Preliminary results in a recent study have demonstrated significant improvement in cognition associated with hearing aid use in older adults (26). Taken together, these findings suggest that early auditory rehabilitation may prevent cognitive decline. Implementing screening tools such as SHOEBOX QuickTest in memory clinics could be one strategy to increase use of hearing aids in patients with cognitive impairment, which may lessen further cognitive decline.

Despite the association between hearing loss and cognitive decline there is a paucity of literature evaluating the reliability of objective hearing loss screening tools in this population. Our study provides preliminary evidence that rapid, objective hearing screeners such as SHOEBOX QuickTest can reliably be used to screen for hearing loss in individuals with mild cognitive impairment. These types of self-administered objective screeners do not require expertise in hearing testing and provide immediate, actional able results (e.g., individual should be referred for a complete audiological evaluation) reducing some potential barriers to implementation. Further research is needed to explore the implementation of hearing screening within memory clinic programs.

## Conclusion

SHOEBOX QuickTest is a valid and accurate hearing loss screening tool for individuals with cognitive impairment. Implementing this screening tool in memory clinics can not only aid in a timely diagnosis of hearing loss, but it can also assist physicians in providing a better assessment of cognitive impairment by ruling out hearing loss as a confounding variable.

## Data Availability Statement

The raw data supporting the conclusions of this article will be made available by the authors, without undue reservation.

## Ethics Statement

The studies involving human participants were reviewed and approved by Bruyère Research Institute. The patients/participants provided their written informed consent to participate in this study.

## Author Contributions

AF designed the methodological approach, collected the data, and supervised the findings and revised the final manuscript. SG performed the data analysis. SG, AMF, MB, and AF wrote the article. All authors contributed to the article and approved the submitted version.

## Funding

Trillium Health Partners (Trillium) has a vision to develop a pan-Canadian integrated health network (CAN Health) that would facilitate the adoption of Canadian developed technology solutions and break through the historic challenges that health and life sciences companies have faced, enabling them to scale and grow in Canada. Government of Canada funded this project; Ottawa Audiology Services Clinic, Jean Dallaire for conducting the audiology tests. SHOEBOX Inc. CHEO (funds held by MB) covered open access journal costs.

## Conflict of Interest

MB is the Chief Medical Officer at SHOEBOX Ltd. AMF is the Manager, Research and Data Analytics at SHOEBOX Ltd. The remaining authors declare that the research was conducted in the absence of any commercial or financial relationships that could be construed as a potential conflict of interest.

## Publisher's Note

All claims expressed in this article are solely those of the authors and do not necessarily represent those of their affiliated organizations, or those of the publisher, the editors and the reviewers. Any product that may be evaluated in this article, or claim that may be made by its manufacturer, is not guaranteed or endorsed by the publisher.
